# Troponin, NT-proBNP and postoperative atrial fibrillation in a prospective cohort undergoing coronary artery bypass surgery

**DOI:** 10.1038/s41598-025-12122-3

**Published:** 2025-08-07

**Authors:** Amelie H. Ohlrogge, Ferdinand Seum, Korbinian Ruhland, Francisco M. Ojeda, Christin S. Börschel, Simon Pecha, Stefan Blankenberg, Tanja Zeller, Renate B. Schnabel

**Affiliations:** 1 Department of Cardiology, University Heart and Vascular Center Hamburg, Hamburg, Germany; 2 German Center for Cardiovascular Research (DZHK), Partner Site Hamburg/Kiel/Lübeck, Hamburg, Germany; 3 Department of Cardiovascular Surgery, University Heart and Vascular Center Hamburg, Hamburg, Germany; 4 University Centre of Cardiovascular Science, University Heart and Vascular Center Hamburg, Hamburg, Germany; 5 University Heart Centre Hamburg-Eppendorf, Building O70, Martinistrasse 52, 20246 Hamburg, Germany

**Keywords:** Postoperative atrial fibrillation, NT-proBNP, High-sensitive troponin T, High-sensitive troponin I, Biomarker, Risk prediction, Predictive markers, Atrial fibrillation

## Abstract

Troponin and N-terminal pro B-type natriuretic peptide (NT-proBNP) are biomarkers of cardiac damage and myocyte stretch. The existing evidence on the predictive value of preoperative high-sensitive Troponin and NT-proBNP concentration for the onset of postoperative atrial fibrillation (POAF) is inconsistent. Therefore, we aimed to assess these biomarkers as predictors for POAF in a prospective observational cohort study of patients without atrial fibrillation undergoing coronary artery bypass graft surgery (CABG). We analysed 423 patients with a median age of 66.3 years, 15.1% were women. About a third of these patients (*N* = 135, 32.4%) developed POAF. The median concentration of (25th, 75th percentile) high-sensitive Troponin at baseline was 11.8 (5.6, 42.7) ng/l in the POAF group and 11.4 (5.2, 37.0) ng/l in the group without POAF, median NT-proBNP was 255 (131, 621) ng/l in the POAF group and 184 (91, 497) in the group without POAF. In uni- and multivariable analyses neither biomarker showed statistically significant associations with POAF. These findings add further neutral data to the inconsistent results found in the current literature and mandate the search for better clinical or biomarker information to assess the risk of this common complication of CABG surgery.

## Introduction

Postoperative atrial fibrillation (POAF) is a common but serious complication after both cardiac and non-cardiac surgery. It occurs in about 25% of patients undergoing cardiac surgery^1^. POAF increases the risk of both mortality and morbidity such as the occurrence of stroke, heart failure and intensive care unit stay and overall hospitalisation^2,3^. POAF also increase treatment costs and long-term rate of complications^4^.

Prediction of atrial fibrillation (AF) in general and POAF with clinical factors and biomarkers is needed to identify individuals at a high risk, with the aim to possibly prevent, monitor and treat the arrhythmia early and thus reduce morbidity and mortality. The perioperative phase entails distinct characteristics that distinguish POAF from AF in the general population. Though not fully understood, these differences comprise cardiac damage inflicted by surgery itself but also altered hemodynamics, electrolyte imbalances, autonomic dysregulation and inflammation induced by surgery and the postoperative phase. The pre-operative characteristics of the patient also plays a role, e.g. age or heart failure^5^. However, prediction of POAF remains imprecise.

Different biomarkers have been proposed and tested for the prediction of (postoperative) AF. Most of these are markers of inflammation or cardiac stress and damage. Of the latter, N-terminal pro brain natriuretic peptide (NT-proBNP) and different troponin subunits (high-sensitive cardiac troponin T and I, hs-cTnT and hs-cTnI) have been examined for the prediction of AF and POAF. NT-proBNP is a natriuretic peptide hormone that is released by cardiac myocytes mostly stimulated by stress on the cardiac walls. It is most commonly used in the diagnosis of initial and worsening of heart failure with and without reduced ejection fraction^6^. Troponin is a structural protein complex which consists of the subunits I, T and C and is exclusively found in the cardiomyocytes. Its detection in the peripheral blood is sensitive and specific for myocardial injury, which has rendered troponin one of the leading biomarkers in the diagnostic of acute coronary syndrome^7^.

We hypothesized that elevated preoperative troponin levels would be associated with an increased risk of POAF and compare it to NT-proBNP. We aimed to examine this hypothesis in a large, prospective cohort of more than 400 stable patients free of AF undergoing CABG.

## Methods

### Study cohort

The Atrial Fibrillation in High-Risk Individuals-Biopsy (AFHRI-B) study is an ongoing, prospective, monocentric cohort study designed to improve POAF risk prediction. AFHRI-B is a sub-study of the clinical cohort study (CCS) conducted at the University Heart and Vascular Centre Hamburg (Germany). We included patients who were 18 years or older undergoing CABG with the support of a heart-lung machine. Individuals who did not have sufficient knowledge of the German language skills to understand the informed consent forms and to participate in the interview were excluded. In addition, individuals in emergency situations or with acute myocardial infarction were excluded. We focused on AF not related to severe heart valve disease and thus excluded individuals with planned valve surgery or high-grade valvular disease. Participation in the study was voluntary. Written informed consent was obtained from all participants. The conduct of this study was approved by the local Ethics Committee of the Medical Association Hamburg (Ärztekammer Hamburg). All methods were carried out in accordance with the relevant guidelines and regulations.

### Data collection

Baseline data was collected in form of an interview using a detailed questionnaire, including information on pre-existing conditions, medication, family history, lifestyle, and cardiovascular risk factors. Baseline information was supplemented by a review of the electronical medical record. Blood was taken from the patients before surgery and stored at − 80 °C. In order to collect postoperative data, questionnaires were mailed to the participants and a standardized telephone follow-up interview took place approximately 30 days after study inclusion. In this interview, changes in wellbeing and habits as well as newly diagnosed cardiovascular diseases, including AF, were recorded. POAF was defined as any new AF episode during the 30-day postoperative period with irregular RR-intervals at the absence of p-waves in an ECG of at least 30 s. For each study participant, all electrocardiograms available in the electronic medical record were analyzed by two experienced investigators. In the case of discrepancies in the diagnosis of AF a third cardiologist or electrophysiologist was consulted. In addition, patients were continuously monitored by telemetry throughout their postoperative hospital stay, which on average lasted six to eight days. In the case of POAF observed on telemetry a 12-lead-ECG was obtained to confirm the diagnosis. During medical rehabilitation patients were not continuously monitored. 12-Lead-ECGs were routinely obtained at least at admission, discharge and additionally at the onset of new symptoms or at the discretion of the treating physicians. Further information on the course of the postoperative treatment were obtained from the discharge report, rehabilitation discharge letters and from the electronic medical record. The primary outcome was newly diagnosed, postoperative AF (POAF). Due to a transition in routine laboratory measurements in May 2020, high sensitivity measured troponin T levels were determined using an Elecysy assay (Roche Diagnostics, Basel, Switzerland) for the first 343 patients, from then on high sensitivity measured troponin I levels were determined in 65 patients using the Atellica IM High-Sensitivity assay (Siemens Healthineers, Erlangen, Germany). NT-proBNP levels were measured using the Elecsys proBNP II IVD assay (Roche Diagnostics, Rotkreuz, Switzerland).

### Statistical analysis

The statistical analysis was carried out using R version 4.3.2. Logistic regression models were set up with POAF as the dependent variable and compared with each other using the Akaike information criterion. The logit function was selected and used as the link function. A linear relationship between the log odds of POAF and the logarithmic troponin values was assumed. All standard assumptions were checked and are met. For the logistic regression analyses both biomarkers were log-transformed. Troponin I concentrations adjusted to fit the distribution of Troponin T concentrations with a constant divisor before log-transformation.

## Results

After excluding patients with prevalent AF, *n* = 423 remained for analysis. Troponin measurements were available in 408 patients, NT-proBNP measurements in 411 patients. The cohort had a median age of 66.3 years, 84.9% were male. The prevalence of classical cardiovascular risk factors was high, 92.0% had hypertension, 31.0% diabetes, 74.5% were smokers, median BMI was 27.8 kg/m^2^. Median hs-cTnT was 11.0 ng/l, median hc-cTnI was 18.0 n/l, median NT-proBNP 219.8 ng/l. 135 patients (32.4%) of patients developed POAF during follow-up. Patients who later developed POAF were significantly older with higher total and LDL cholesterol levels at baseline. These patients had marginally higher levels of troponin (both hs-cTnT and hs-cTnI) and NT-proBNP, though without statistical significance. Hypertension, diabetes, BMI and smoking differed little between the POAF and no POAF group. In patients with additional surgery (e.g. additional valve surgery) POAF was significantly more common than in those with bypass surgery only. Slightly more women than men developed POAF, again without reaching statistical significance (Table 1).

**Table 1:** Baseline characteristics.

	All	POAF	No POAF	*P*-value for difference*
Postoperative AF No. (%)	135 (32.4)	135 (100)	0 (0)	–
Age (years)	66.3 (58.9, 72.8)	70.0 (64.6, 75.6)	63.7 (57.4, 71.1)	< 0.001
Men No. (%)	359 (84.9)	108 (80.0)	251 (87.2)	0.06
Hypertension No. (%)	389 (92.0)	123 (91.1)	266 (92.4)	0.66
Diabetes No. (%)	131 (31.0)	42 (31.1)	89 (30.9)	0.97
Body mass index (kg/m^2^)	27.8 (24.9, 30.9)	27.5 (25.0, 30.9)	27.8 (24.8, 30.9)	0.82
Smoking No. (%)	313 (74.5)	100 (74.1)	213 (74.7)	0.88
HDL-cholesterol (mg/dl)	45.0 (36.0, 53.0)	44.0 (36.0, 54.0)	37.0 (32.0, 44.0)	0.83
LDL-cholesterol (mg/dl)	98.5 (75.8, 127.8)	110.5 (82.0, 136.0)	98.0 (68.0, 135.0)	0.02
Total cholesterol (mg/dl)	175.0 (145.0, 197.0)	189.0 (154.0, 220.0)	152.0 (124.5, 198.5)	0.01
Additional surgery No. (%)	32 (7.6)	17 (12.6)	15 (5.2)	0.01
hs-cTnT(ng/l)	11.0 (5.4, 32.0)	11.1 (5.7, 29.2)	10.7 (5.9, 34.9)	0.61
hs-cTnI (ng/l)	18.0 (5.8, 163.8)	57.0 (5.0, 550.0)	14.0 (6.5, 118.0)	0.47
NT-proBNP (ng/l)	219.8 (97.9, 565.5)	254.6 (130.6, 621.2)	184.0 (91.3, 497.0)	0.61

For categorical variables, percentages are displayed in brackets. For continuous variables the median is displayed, with the 25th and 75th percentile displayed in brackets. *p-value for difference between POAF and no POAF group.

**Fig. 1:**
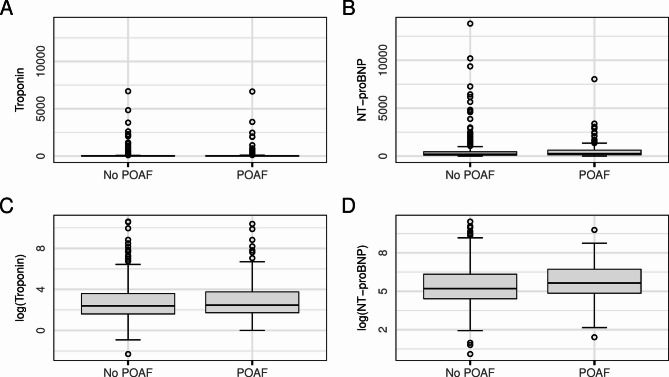
Boxplots for the distribution of Troponin (Troponin T and I adjusted, Panel **A** and **C**) and NT-proBNP (Panel **B** and **D**) in absolute values (Panel **A** and **B**) and log-transformed (Panel **C** and **D**). In Panel **A** a total of five outliers are not displayed (three individuals without POAF, two individuals with POAF). POAF, postoperative atrial fibrillation.

Both biomarkers showed a logarithmic normal distribution and were thus log-transformed for the following analysis. Taking the 99th percentile as cut-off for both hs-cTnT and hs-cTnI, *n* = 90 patients (21.3%) had an elevated troponin. Figure 1 shows the distribution of troponin and NT-proBNP values in the cohort, both in absolute values and log-transformed.

**Fig. 2:**
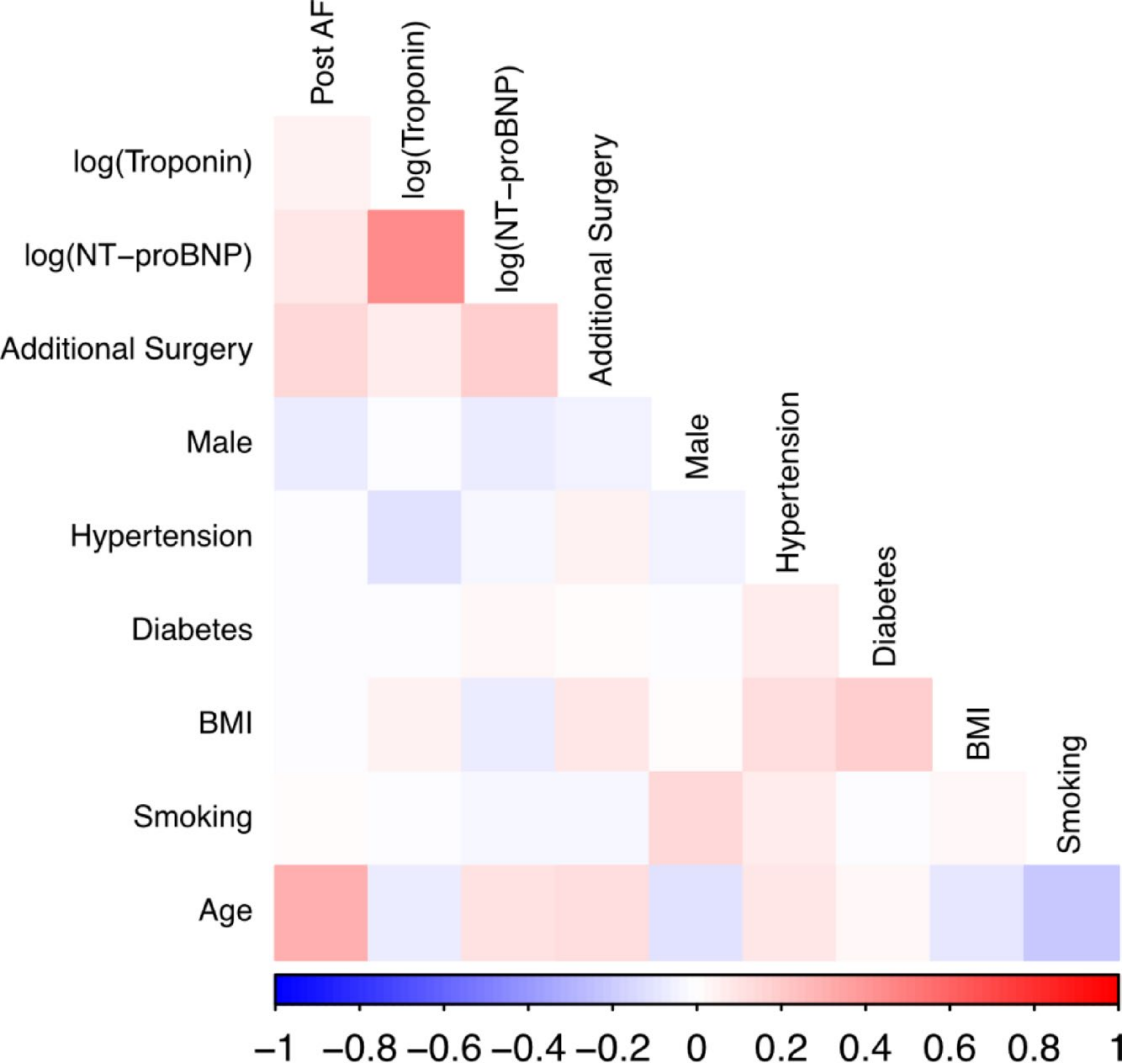
Heatmap plot displaying the correlation of biomarkers and cardiovascular risk factors with each other and POAF.

The log-transformed biomarkers strongly positively correlated with each other (*r* = 0.44 between log(Troponin) and log (NT-proBNP)). Other strong positive correlations were observed between age and POAF (*r* = 0.31), BMI and diabetes (= 0.19), smoking and male sex (0.14), BMI and and hypertension (*r* = 0.13) and log(NT-proBNP) and POAF (*r* = 0.09). Strong negative correlations were observed between age and smoking (*r*=-0.22), male sex and age (*r*=-0.12) (Fig. 2).

In an univariable logistic regression log(Troponin) was marginally positively associated with POAF, with an odds ratio (OR) of 1.05 and a non-significant p-value of 0.38 (Model 1). In a multivariable model including age, sex and additional surgery the OR of log(Troponin) was 1.07 with a non-significant *p*-value of 0.25 (Model 2). These results did not significantly differ when further adjusting the models for hypertension, diabetes, BMI and smoking (Model 3). Age was positively and significantly associated with increased risk of POAF in both models (OR of 1.08 and 1.09 respectively, *p* < 0.001 for both models) (Table 2).

**Table 2:** Odds ratios with 95% confidence intervals (CI) for log(troponin), and POAF.

Variable	Model 1	Model 2	Model 3
	OR (95% CI)	OR (95% CI)	OR (95% CI)
log(Troponin)	1.05	1.07	1.07
	(0.94–1.17)	(0.95–1.20)	(0.95–1.20)
	*p* = 0.38	*p* = 0.25	*p* = 0.30
Age		1.08	1.09
		(1.05–1.09)	(1.06–1.19)
		*p* < 0.001	*p* < 0.001
Male sex		0.83	0.77
		(0.46–1.50)	(0.42–1.40)
		*p* = 0.53	*p* = 0.39
Additional surgery		2.44	2.4
		(1.06–5.63)	(1.02–5.63)
		*p* = 0.04	*p* = 0.044
Hypertension			0.63
			(0.28–1.44)
			*p* = 0.27
Diabetes			1.00
			(0.61–1.64)
			*p* = 0.99
Body mass index			1.01
			(0.97–1.06)
			*p* = 0.58
Smoking			1.55
			(0.91–2.64)
			*p* = 0.11

Model 1 is unadjusted, model 2 is adjusted for age, sex and additional surgery, model 3 additionally includes hypertension, diabetes, body mass index and smoking in addition to model 2.

Using the same model with log(NTproBNP) instead of log(troponin) yielded comparable results. In univariable logistic regressions log(NTproBNP) had a higher OR than log(troponin) of 1.16, with a p-value of 0.06, thus failing statistical significance (Model 1). In model 2 (adjusted for age, sex and additional surgery) and model 3 (further adjusted for hypertension, diabetes, BMI and smoking) OR were 1.07 (95% CI 0.90–1.26, *p* = 0.46) and 1.06 (95% CI 0.89–1.25, *p* = 0.51), respectively. Similar to the models with log(troponin), age significantly increased risk of POAF (Table 3).

**Table 3:** Odd ratios with 95% confidence intervals in brackets for log(NT-proBNP), classical cardiovascular risk factors and POAF.

Variable	Model 1	Model 2	Model 3
	OR (95% CI)	OR (95% CI)	OR (95% CI)
log(NT-proBNP)	1.16	1.07	1.06
	(0.99–1.35)	(0.90–1.26)	(0.89–1.25)
	*p* = 0.06	*p* = 0.46	*p* = 0.51
Age		1.07	1.08
		(1.05–1.10)	(1.05–1.11)
		*p* < 0.001	*p* < 0.001
Male sex		0.78	0.74
		(0.43–1.43)	(0.41–1.35)
		*p* = 0.43	*p* = 0.33
Additional surgery		2.57	2.56
		(0.90–1.26)	(1.10–6.00)
		*p* = 0.03	*p* = 0.03
Hypertension			0.63
			(0.28–1.41)
			*p* = 0.26
Diabetes			0.96
			(0.58–1.57)
			*p* = 0.86
BMI			1.01
			(0.97–1.06)
			*p* = 0.58
Smoking			1.44
			(0.85–2.44)
			*p* = 0.17

Model 1 includes only log(NT-proBNP), model 2 additionally included age, sex and additional surgery. Model 3 includes hypertension, diabetes, BMI and smoking in addition to model 2.

A post-hoc power analysis was conducted. For a power 0.99, the upper bound for Cohen’s f^2^ is 0.047, suggesting that the study was sufficiently powered to detect even small-to-moderate effects.

## Discussion

We present data from a monocentric prospective cohort of patients undergoing coronary artery bypass graft surgery without relevant valvular disease. In this cohort, neither preoperative troponin (T and I) nor NT-proBNP were associated with a significantly increased risk of POAF in multivariable-adjusted analyses. Preoperatively, both biomarkers had marginally higher levels in patients who later developed POAF than in those who did not, without reaching statistical significance.

These findings add neutral results to the inconsistent literature for both biomarkers in this context. The interpretation of these heterogenous results is further complicated by substantial variations in methodology between these studies: different cohorts, different types of surgery (isolated CABG, isolated valve surgery or all cardiac surgery), different timepoints of sampling (pre- or postoperative), different mode of follow-up with wide ranges in POAF prevalence (6–43.9%^8,9^), and different troponin subunits (hs-cTnT and hs-cTnI). Most studies are relatively small with < 200 patients. We have identified only one study that analysed NT-proBNP^10^ and three studies analysing troponin^10–12^ with a sample size larger than in our analysis, though only two of the latter analysed high-sensitive troponin^10,12^.

A recent meta-analysis of 10 studies, with almost 2,000 patients found higher preoperative NT-proBNP levels in patients undergoing CABG who later developed POAF compared to the sinus rhythm group, both for isolated CABG and combined or mixes cardiac surgery cohorts^13^. However, at least one study reporting no significant association of pre- and postoperative NT-proBNP levels with POAF was not included in this meta-analysis^14^. It is further worth noting that in the largest study included in this meta-analysis, the OPERA (Omega-3 Fatty Acids for Prevention of Post-Operative Atrial Fibrillation trial) trial, higher levels of preoperative NT-proBNP were observed in patients who later developed POAF but were not independently associated with the onset of POAF^10^.

The largest study to date, a retrospective analysis of 3,148 patients found elevated perioperative troponin T levels associated with increased risk of POAF only in univariable, not independently in multivariate analyses, and low discriminatory ability (area under the curve values 0.52–0.55)^11^. In another large study, including 2,421 patients undergoing isolated CABG preoperative, not perioperative, hs-cTnT was significantly associated with an increased risk of POAF, though with a low accuracy (area under the curve 0.625)^12^. Similar results were found in 100 patients undergoing cardiac surgery^14^. In contrast, two rather small studies with 95 and 38 patients undergoing CABG found only postoperative, not preoperative hs-cTnT to be a predictor of POAF^15,16^. Preoperative hs-cTnT levels were higher in patients who developed POAF in one study^17^. In the OPERA trial hs-cTnT was not independently associated with the risk of POAF in 562 patients undergoing cardiac surgery^10^. Among 137 patients undergoing cardiac surgery hs-cTnI levels were higher in the POAF group, but hs-cTnI was not an independently associated risk factor^18^. In two studies, postoperative troponin I showed no significant difference between between the group that developed POAF an those who did not in 156 and 81 patients undergoing CABG^19,20^.

As NT-proBNP and troponin are markers of myocardial stress and damage, one could argue that it is pathophysiologically plausible that higher levels are associated with an increased risk of POAF. Outside of the postoperative setting, NT-proBNP is an established and robust predictor of AF^21,22^ whereas troponin is less potent in predicting the onset, but rather the complications and outcomes in patients with AF^23,24^. In general, these biomarkers are influenced by several confounders such as age and kidney function that also influence risk of (postoperative) AF. Additional clinical features such as coronary artery disease (CAD) or valvular disease that lead to cardiac surgery in POAF cohorts also increase both biomarkers but may also independently increase the risk of POAF. Therefore, the ability of troponins clearly is limited in the setting of stable CABG patients.

Considering this inconsistent but also methodologically heterogenous literature we are unable to explain our neutral findings with certainty. It is conceivable that the effect size is too small, at least in the specific settings of our cohort, to be detected in this sample size. On the other hand, our cohort is among the largest that can be found in literature as of now. Since statistically significant results have been detected in smaller cohorts than ours it could have been expected that these results in a cohort with more than 400 patients replicate. While it is possible that previous, positive findings with small sample sizes have been subject to a type I error perpetuated by publication bias, it is conceivable that our results are subject to a type II error (false negative result), and we failed to detect an actual effect. However, despite different troponin tests, strong associations would have been detected. Based on the results of our post-hoc power analysis it is highly unlikely that the negative results in the study presented here are a type II error caused by an inadequate sample size. A plausible explanation of our null findings in line with higher quality studies published recently, is that the biomarkers of cardiac injury and stress are elevated by the nature of the underlying diseases and thus discriminatory ability is impaired. Both, strict publishing of neutral or negative results and more large-scale studies are needed to clarify the inconsistency of the currently available data.

## Conclusions

In our prospective cohort of over 400 patients free of high-grade valvular disease undergoing on-pump CABG the biomarkers high-sensitive troponin (T and I) and NT-proBNP were not strongly associated with the risk of POAF. These findings add further neutral data to the inconsistent results found in current literature and mandate the search for better clinical or biomarker information to assess the risk of this common complication of CABG surgery.

## Limitations and strengths

A major limitation in this analysis is the utilization of two different troponin subunits (hs-cTnI and hs-cTnT) within the cohort. Both subunits correlate strongly with each other and both have excellent diagnostic accuracy, though with different accuracy in the early hours of acute myocardial infarction^25^. However, our patients presented in a stable clinical condition. This is underlined by the fact that almost 80% of our patients had a troponin below the 99th percentile, regardless of the comorbidity burden and severe CAD that necessitated CABG. In stable CAD patients, both troponin subunits are comparable in their association with future cardiovascular events^26^ and prediction of all-cause mortality^27^. Further, our study is monocentric, and patients with high-grade valvular disease were excluded. While the latter limits confounding, it also restricts generalizability to all cardiac surgery patients.

A major strength of this analysis lies in the comparatively large sample size of this homogeneous cohort. During pre-operative 24 h monitoring we excluded high burden prevalent AF. As discussed above there are few comparable analyses cohorts of in this sample size or greater^10–12^. Also, it is a prospective, thoroughly characterized and followed-up cohort.

## Data Availability

The datasets used and/or analysed during the current study available from the corresponding author on reasonable request.

## Abbreviations

AF: Atrial fibrillation
BMI: Body mass index
CABG: Coronary artery bypass graft
CAD: Coronary artery disease
NT: proBNP-N-terminal pro brain natriuretic peptide
hs: cTnI-high-sensitive cardiac troponin I
hs: cTnT-high-sensitive cardiac troponin T
OR: Odds ratio
POAF: Postoperative atrial fibrillation
